# Retraction statement: Design, development, and evaluation of a registry system for hyperbaric ‎oxygen therapy

**DOI:** 10.1002/hsr2.1456

**Published:** 2023-07-25

## Abstract

The following article, published online on {First published: 08 August 2022} in Wiley Online Library {https://onlinelibrary.wiley.com/doi/full/10.1002/hsr2.768} has been retracted by agreement between the journal's Editor‐in‐Chief, Esmaeil Mehraeen and John Wiley & Sons Ltd. The retraction has been agreed given the journal has received evidence confirming that the peer review process of this paper was manipulated. As a result, the conclusions reported in the article are not considered reliable.
